# Patient-reported outcome measures developed for non–cystic fibrosis bronchiectasis may be applied to cystic fibrosis bronchiectasis

**DOI:** 10.1186/s12955-026-02546-4

**Published:** 2026-05-13

**Authors:** Patrick A. Flume, Robert J. Nordyke, Donald Han, Ashok Jha, Gina Nicholson, John Devin Peipert

**Affiliations:** 1 Medical University of South Carolina, Charleston, SC USA; 2 Beta6 Consulting Group, Los Angeles, CA USA; 3 Boehringer Ingelheim Pharmaceuticals, Inc., Ridgefield, CT USA; 4 Boehringer Ingelheim International GmbH, Ingelheim am Rhein, Germany; 5 Centre for Patient Reported Outcomes Research, University of Birmingham, Edgbaston, Birmingham UK

**Keywords:** Cystic fibrosis bronchiectasis, Health-related quality of life, Patient-reported outcome measures, Symptoms

## Abstract

**Background:**

Bronchiectasis (BE) is a chronic lung disease that impacts health-related quality of life (HRQoL). BE has historically been separated into cystic fibrosis (CF)-related BE (CFBE) and non–CF-related BE (NCFBE [where NCFBE is etiologically heterogeneous or idiopathic]), despite both sharing similar pathophysiology. Patient-reported outcome measures (PROMs) assessing BE symptoms and impacts on HRQoL have largely been developed and validated in NCFBE. This study assessed whether PROMs developed for NCFBE reflect HRQoL experiences of people with CFBE.

**Methods:**

A preliminary conceptual model of symptoms and HRQoL impacts most relevant to people with CFBE was developed based on expert opinion, the content of existing PROMs, and previous qualitative research. Items from 11 existing PROMs were mapped to this preliminary conceptual model. A focus group discussion guide was created from the preliminary conceptual model. US participants aged ≥ 19 years with a self-reported diagnosis of CF and BE were eligible for participation in one of two focus groups (December 2023 or January 2024). Focus groups were recorded and transcribed verbatim, and thematic analysis (ATLAS.ti Web) was used to identify recurring themes from the discussions. Final themes were mapped back to the preliminary conceptual model to identify potential gaps between existing PROMs and relevant HRQoL impacts.

**Results:**

A total of 19 people with CFBE participated in the virtual focus groups. Most participants were female (63%) and non-Hispanic White (95%), ranging from 22 to 67 years of age. Focus group discussions revealed five primary themes: Emotional Symptoms, Physical Function, Physical Symptoms, Social Health, and Treatment Burden. A total of 242 unique mentions of BE-related symptoms and impacts on HRQoL were mapped to the model. Generally, concerns about symptoms and HRQoL impacts expressed by participants were consistent with the model. Potential discrepancies included feelings of loss of control, sinusitis, and hemoptysis. Although not included in the model, loss of control likely overlapped with anxiety or treatment burden. Hemoptysis, although raised during focus group discussions, was not captured in the model, but is captured in several PROMs.

**Conclusions:**

Measures developed to assess patient‑reported outcomes in NCFBE may be extended to CFBE, pending cognitive and psychometric validation.

**Supplementary Information:**

The online version contains supplementary material available at 10.1186/s12955-026-02546-4.

## Background

Bronchiectasis (BE) is a chronic inflammatory lung disease with considerable etiologic heterogeneity [1–3]. Hallmarks of BE include irreversible dilation of the bronchi, sputum production, and cough [4, 5]. BE pathogenesis and progression typically result from an interplay of chronic neutrophilic inflammation, persistent infection, impaired mucociliary clearance, and progressive structural lung damage [1, 2, 6]. These interactions can lead to frequent pulmonary exacerbations (PExs) in people with BE [1]—serious events associated with a high burden of daily symptoms and poor health-related quality of life (HRQoL), often leading to hospitalizations and mortality [5, 7].

BE imposes a considerable burden on patients, caregivers, and healthcare systems [8–10]. People with BE often report adverse impacts on HRQoL [11, 12] (with lower HRQoL compared with the general population [13]) and exercise intolerance [14, 15]. Small studies have also shown that BE has a negative impact on a person’s social life and hobbies [11], and that people with BE are at higher risk of mental health problems [16]. While the underlying pathophysiology of BE is shared across many etiologies, people with cystic fibrosis (CF) who have BE (CFBE) have been historically grouped as a distinct disease population. Consequently, BE is often categorized into CFBE versus non-CFBE (NCFBE, where BE arises from other etiologies or an unknown cause). Despite CFBE sharing a common disease pathophysiology with BE of other etiologies [17], it is possible that the two populations have different lived experiences. The burden of illness in CF (largely due to BE) begins early [18, 19], whereas BE unrelated to CF typically develops later in life [20, 21]. Additionally, although similarities in disease pathophysiology may lead to similarities in disease outcomes, it is unclear if these similarities translate into overlapping symptoms and HRQoL impacts between people with NCFBE versus CFBE.

Due to the profound effect of BE on affected individuals, several patient-reported outcome measures (PROMs) have been developed to capture BE symptoms and impacts on HRQoL. However, due to the two populations’ historical separation, the original qualitative development of existing PROMs for BE has generally not explicitly included a CFBE population, and many studies validating these PROMs have largely been conducted in NCFBE populations [15, 22–24]. Thus, this study aimed to assess whether the HRQoL experience in adults with CFBE can be captured by existing PROMs that were primarily developed in people with NCFBE. This article is written in accordance with COnsolidated criteria for REporting Qualitative research (CORE-Q) guidelines [25]. 

## Methods

Three components were used to assess whether existing PROMs capture HRQoL impacts that are most relevant to people with CF: the development of a preliminary conceptual model, focus group discussions, and alignment with the preliminary conceptual model using thematic analysis.

### Development of the preliminary conceptual model

A preliminary conceptual model of the symptoms and HRQoL impacts most relevant to people with BE (inclusive of CFBE) was created. This model was based on expert clinical opinion, the content of existing PROMs in BE, and previous qualitative research in people with BE. This was further expanded by a targeted review of available literature on PROMs for BE, forming the set of measures evaluated in this study. The review process focused on the instruments currently used to measure HRQoL in people with BE and aimed to identify other PROMs/HRQoL concepts from qualitative research in BE or clinical development programs.

The supporting literature review consisted of three components: (1) the assessment of relevant PROMs from peer-reviewed literature, (2) the assessment of PROMs used in registered BE clinical trials, and (3) a summary of the prescribing information for selected analog products for severe respiratory conditions. Details are provided in Supplementary Material 1.

The preliminary conceptual model was used to characterize the relevant concerns reflected in individual items in the 11 PROMs (nine multi-item questionnaires and two daily symptom diaries) evaluated in this study (Table 1).

**Table 1:** Existing PROMs used for the development of the preliminary conceptual model

PROM		Publication date	Industry sponsor	Patient population involved in development
Multi-dimension PROMs	BHQ [26]	2017	None	NCFBE
	BIM [27]	2020	EMBARC2	NCFBE
	CAAT [28]	2009	GlaxoSmithKline	COPD
	CASA-Q [29]	2008	Boehringer Ingelheim	COPD
	CFQ-R [30]	2009	Gilead	CF
	CRS-PRO [31]	2020	None	CRS
	LCQ [32]	2003	None	Cough
	QOL-B [33]	2014	Gilead	NCFBE
	SGRQ [34]	1992	None	COPD
Daily diaries	BED [35]	2023	AstraZeneca	NCFBE
	BEST diary [36]	2020	EMBARC2	NCFBE

BED, Bronchiectasis Exacerbation Diary; BEST, Bronchiectasis Exacerbation and Symptoms Tool; BHQ, Bronchiectasis Health Questionnaire; BIM, Bronchiectasis Impact Measure; CAAT, Chronic Airway Assessment Test; CASA-Q, Cough and Sputum Assessment Questionnaire; CF, cystic fibrosis; CFQ-R, Cystic Fibrosis Questionnaire – Revised; COPD, chronic obstructive pulmonary disease; CRS, chronic rhinosinusitis; CRS-PRO, Chronic Rhinosinusitis – Patient-Reported Outcome Measure; LCQ, Leicester Cough Questionnaire; NCFBE, non–cystic fibrosis bronchiectasis; PROM, patient-reported outcome measure; QOL-B, Quality of Life Questionnaire – Bronchiectasis; SGRQ, St. George’s Respiratory Questionnaire

### Patient focus groups

The focus group discussion guide (Supplementary Material 2) was developed by a collaborative eight-person research team, including physicians (respirologists), psychometricians, and outcomes researchers. The discussion guide consisted of questions that sought to understand patients’ respiratory and systemic symptoms, and common problems associated with treatment burden, as well as how these problems affect patients’ day-to-day experiences and feelings.

The focus group setting was chosen because it allowed participants to introduce their own experiences, and react to and build on the impacts expressed by other participants. Semi-structured virtual focus groups with US adults with CF, with a self-reported BE diagnosis, were conducted. Eligible participants were required to have had at least one objective measure of a PEx in the last 12 months prior to the study, involving: (1) a new or changed oral antibiotic; (2) the use of an intravenous antibiotic; or (3) hospitalization to treat the worsening of lung symptoms.

Participants were recruited via convenience sampling in collaboration with Savvy Cooperative [37], a US-based organization that maintains disease-specific panels of people who are willing to be contacted for participation in research. Savvy Cooperative contacted participants based on recruitment criteria and provided the email addresses of those willing to participate in this research. The focus groups, which were 2 h in duration, were conducted by trained researchers (RJN, GN, and DH) who encouraged participants to have open discussions and contribute as they felt comfortable. Information about the discussion moderators is in Supplementary Material 3. Focus groups were conducted via video conferencing (GoTo Meeting platform), but were recorded as audio files (mp3) and transcribed verbatim using the transcription capability of the web-conferencing app. All transcripts were checked independently by two researchers to ensure accuracy following transcription and retention of patient anonymity. Transcripts were not returned to participants. Data saturation was reached if any concerns raised during the second focus group were mentioned only once or twice (considered as having limited impact to participants and thus not relevant enough to necessitate a third focus group).

### Thematic analysis and alignment with the preliminary conceptual model

This content analysis was conducted in two steps. Firstly, thematic analysis [38] was used to identify recurring themes from the focus group discussions, where analysis and transcript coding were conducted using ATLAS.ti Web [39]. Secondly, themes arising from the focus groups were assessed and mapped to the preliminary conceptual model to identify any potential gaps between the HRQoL impacts most relevant to CFBE and any existing PROMs. Detailed information regarding this process can be found in Supplementary Material 4.

## Results

### Preliminary conceptual model

**Fig. 1:**
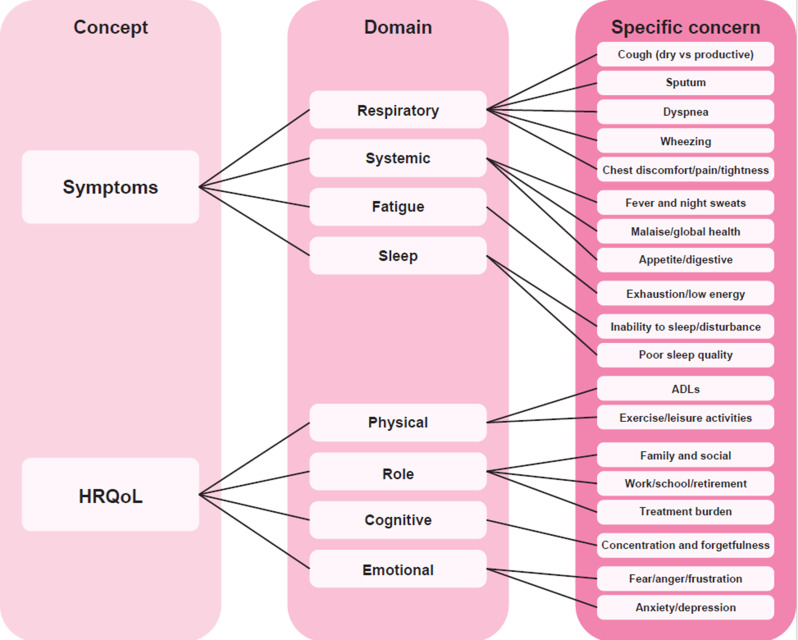
Preliminary conceptual model, developed from existing literature and expert clinical experience. ADL, activity of daily living; HRQoL, health-related quality of life

The preliminary conceptual model, capturing both symptoms and HRQoL impacts, is depicted in Fig. 1. Symptom concerns mainly focused on Respiratory symptoms, but also included potential domains for Systemic symptoms, as well as Fatigue and Sleep problems. HRQoL domains included Physical, Role, and Emotional functioning domains, as well as a Cognitive domain (guided by prior qualitative work) [11, 29, 32].

**Fig. 2:**
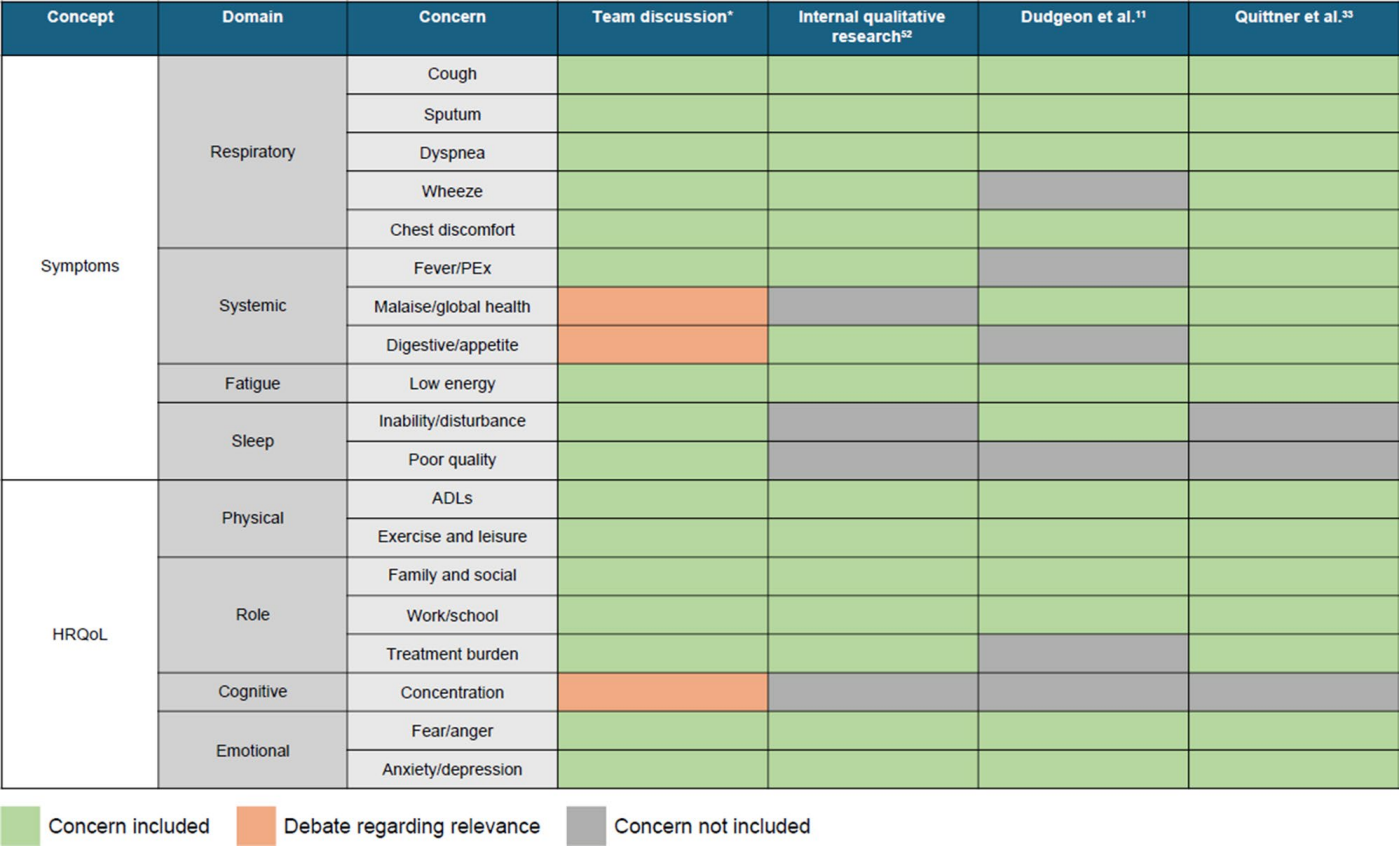
Mapping of existing qualitative research and team discussions to the preliminary conceptual model [11, 33, 52]. ADL, activity of daily living; HRQoL, health-related quality of life; PEx, pulmonary exacerbation. *The research team included pulmonologists, psychometricians, and experts in patient-reported outcomes, clinical trial design, and qualitative research

Concordance between the qualitative research sources and clinical discussions with the preliminary model is shown in Fig. 2. Following this concordance, digestive symptoms and cognitive functioning were excluded from subsequent versions of the preliminary conceptual model (mixed support in qualitative literature).

Of the 11 identified PROMs (Table 1), only five (Bronchiectasis Health Questionnaire, Bronchiectasis Impact Measure [BIM], Quality of Life Questionnaire – Bronchiectasis [QOL-B], and the Bronchiectasis Exacerbation Diary and Bronchiectasis Exacerbation and Symptoms Tool [BEST] diary) [26, 27, 33, 35, 36] used an NCFBE population during original qualitative development. All PROMs initially developed in people with NCFBE were consistent with US Food and Drug Administration-specific guidance on PROMs [40]. Most of the PROMs included questionnaires that were not originally developed in NCFBE populations but have since been used in studies of people with NCFBE. The exception to this is the Cystic Fibrosis Questionnaire – Revised (CFQ-R), which has been psychometrically validated in CF only. However, the QOL-B was based on the earlier CFQ-R, and thus the two PROMs have many questions in common—particularly regarding the Respiratory Symptoms Scale component [30, 33].

**Fig. 3:**
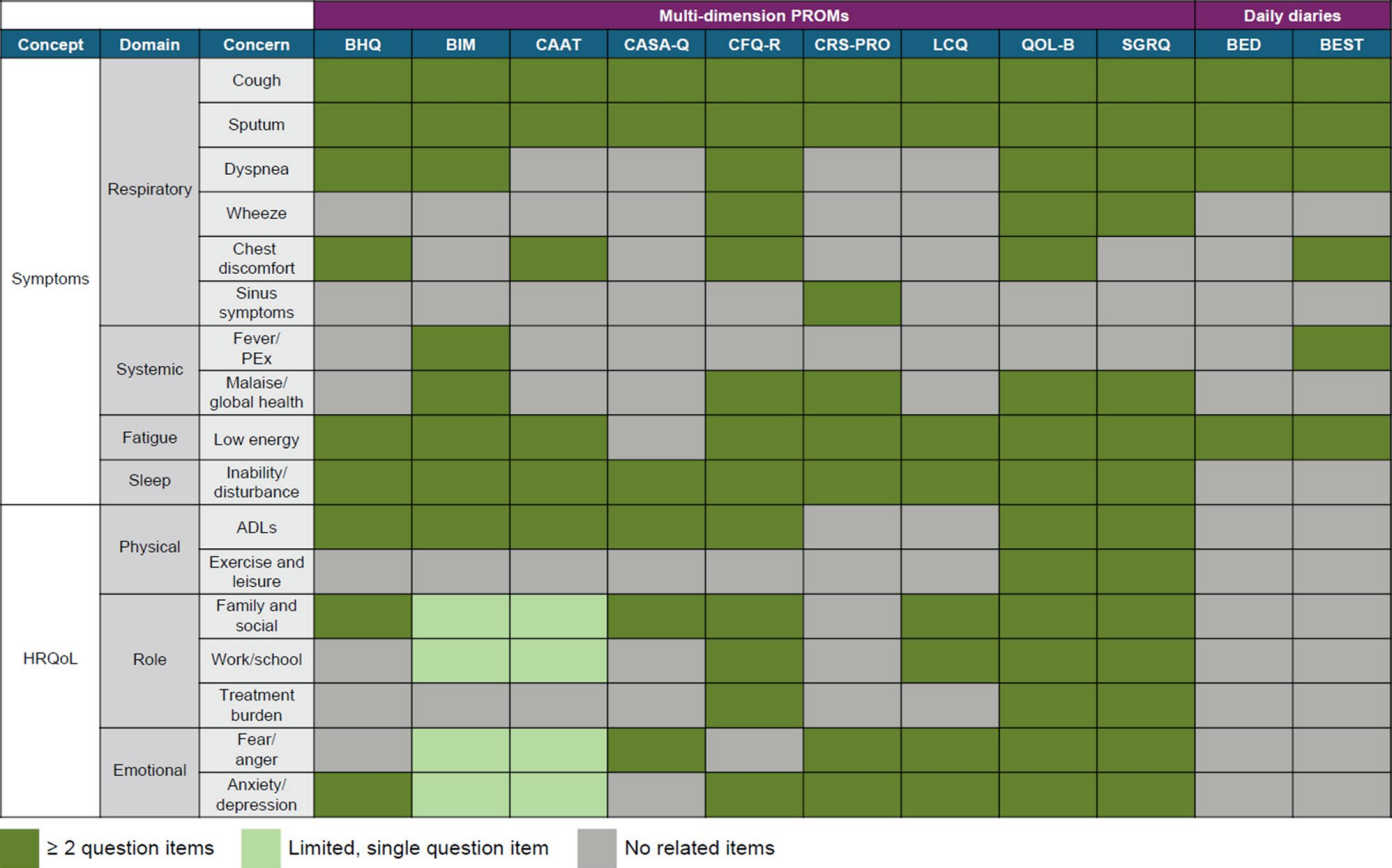
Mapping of the 11 existing PROMs evaluated in this study to the preliminary conceptual model. ADL, activity of daily living; BED, Bronchiectasis Exacerbation Diary; BEST, Bronchiectasis Exacerbation and Symptoms Tool; BHQ, Bronchiectasis Health Questionnaire; BIM, Bronchiectasis Impact Measure; CAAT, Chronic Airway Assessment Test; CASA-Q, Cough and Sputum Assessment Questionnaire; CFQ-R, Cystic Fibrosis Questionnaire – Revised; CRS-PRO, Chronic Rhinosinusitis – Patient-Reported Outcome Measure; HRQoL, health-related quality of life; LCQ, Leicester Cough Questionnaire; PEx, pulmonary exacerbation; PROM, patient-reported outcome measure; QOL-B, Quality of Life Questionnaire – Bronchiectasis; SGRQ, St. George’s Respiratory Questionnaire. The number of items that each PROM contains related to individual HRQoL concerns is represented by color: dark green indicates ≥ 2 question items; light green indicates a single item; and gray indicates that no items are present

Figure 3 presents a mapping of individual items in the included PROMs to the preliminary conceptual model. Many of the included PROMs captured most of the concerns in the framework; however, the St. George’s Respiratory Questionnaire (SGRQ) and QOL-B generally best reflected the concerns in the framework. Of the 17 specific concerns in the preliminary conceptual model, the QOL-B captured 15 concerns, the SGRQ captured 14, the CFQ-R captured 13, the BIM captured 12, and the Chronic Airway Assessment Test (CAAT) captured 10. Of interest, an NCFBE-specific PROM (the QOL-B) captured more topics than a CF-specific PROM (the CFQ-R). Notably, several of the HRQoL concerns in Role and Emotional functioning are measured only by single items in the BIM and CAAT.

### Patient characteristics

A total of 21 participants were recruited, of whom 19 participants engaged in two separate virtual focus groups (*n* = 9 and *n* = 10, respectively). The two additional patients who were recruited were ultimately unable to attend either focus group. The two virtual focus groups were held during December 2023 and January 2024. Table 2 presents an overview of the characteristics of the focus group participants.

**Table 2:** Focus group participant characteristics

Baseline characteristic*		*N* = 19
Gender	Female Male	12 (63) 7 (37)
Age (years)	Mean Range	40 22–67
Race/ethnicity	Black/African American Non-Hispanic White	1 (5) 18 (95)
Education	High school/GED Some college/Associate degree Bachelor’s degree	3 (16) 13 (68) 3 (16)
US Census region	West Midwest Northeast South	6 (32) 5 (25) 6 (32) 2 (11)
CFTR modulator therapy use	None Prior, but now discontinued Current use	3 (16) 3 (16) 13 (68)
Self-reported PEx in the last 12 months	None ≥ 1	9 (47) 10 (53)

CFTR, cystic fibrosis transmembrane conductance regulator; GED, general educational development; PEx, pulmonary exacerbation

*Data presented as *n* (%) unless otherwise specified

### Thematic analysis of focus group discussions

**Fig. 4:**
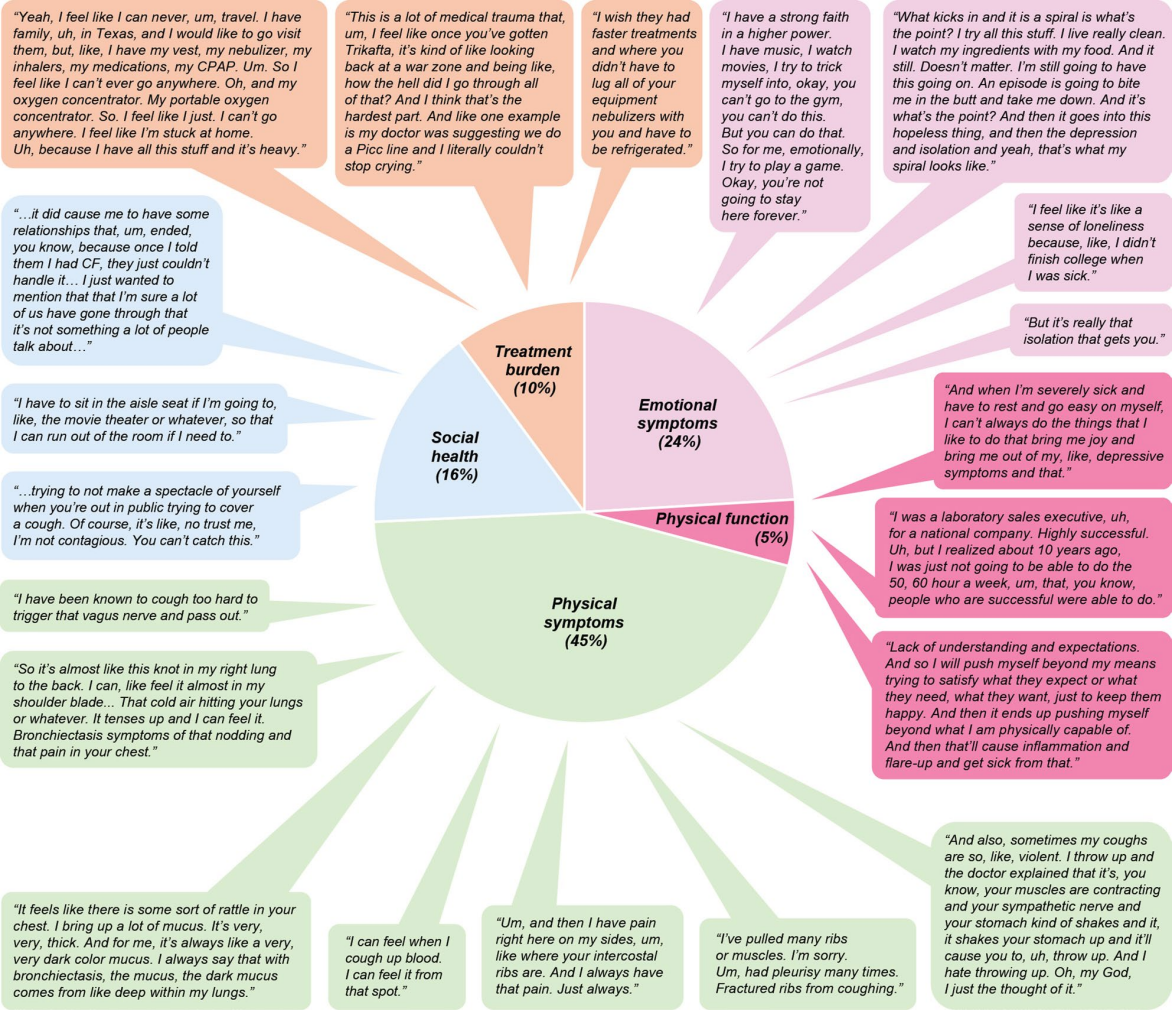
Participant quotes related to the five themes identified during focus group discussions. CF, cystic fibrosis; CPAP, continuous positive airway pressure machine; PICC, peripherally inserted central catheter

Five primary themes of BE symptoms and HRQoL impacts were identified from the focus group discussions (Table 3). Participant quotes relating to these five themes can be seen in Fig. 4.

**Table 3:** Bronchiectasis-related themes emerging from focus group discussions

Theme	Number of mentions, *n* (%)
Emotional impacts	57 (24)
Physical functioning	12 (5)
Physical symptoms	107 (45)
Social health	37 (16)
Treatment burden	24 (10)
**Total**	**237**

#### Emotional impacts

A range of emotional symptoms was reported by nearly all participants. Depression was reported by nearly all participants and anxiety was reported by half of the participants across both focus groups. Fear and anger were reported by a few participants. Additionally, feelings of isolation, hopelessness, or shame were brought up repeatedly across both focus groups.

#### Physical functioning

Participants expressed several general concerns related to physical functioning, impact on daily activities, and work impacts. Examples of these concerns included pushing oneself beyond physical limits to satisfy other people’s wants, being unable to enjoy oneself when sick, and being unable to maintain a job, respectively.

#### Physical symptoms specific to BE

The top physical symptoms that impacted participants’ daily lives were respiratory symptoms of cough, sputum, and chest pain. Wheezing and dyspnea (dyspnea described as “shortness of breath”, trouble with exhaling, and “hard to breathe” by participants) were reported by two participants each, and sinus problems were reported by one participant. Participants were also asked if they had any symptoms that they felt were specific to BE. The presence of mucus and hemoptysis (described as “coughing up blood” by some participants and “hemoptysis” by other participants) as distinguishing symptoms of BE was noted. A few participants also referenced lung or chest pain and tightness that they felt was specific to BE.

#### Social health

Participants with CFBE felt limited in maintaining careers and interacting socially due to the maintenance requirements of the condition, as well as how they appeared to outsiders. Concerns of interest specifically related to BE included coughing in public and having to take medicine prior to eating, which was described as making one feel “socially awkward”. The benefits of a strong social and familial support system were highlighted, as were the negative impacts of minimal social support.

#### Treatment burden

Participants expressed several mechanisms used to manage physical symptoms and emotional impacts, including pre-planning outside engagements, spiritual beliefs, and physical activity. The impacts of having to navigate the medical system were described by multiple participants in both focus groups. Educating providers, knowing who the “good” and “bad” providers in the medical system are, and knowing your body better than anyone else were key subthemes. Two participants also reported on the emotional trauma associated with hospitals or medical centers and having to deal with the residual effects of said trauma.

### Alignment with the preliminary conceptual model

A total of 242 unique mentions of BE-related physical symptoms and impacts on HRQoL (including but not limited to: 70 mentions of respiratory symptoms, 70 mentions of role functioning impacts, and 55 mentions of emotional functioning impacts) were mapped back to the conceptual disease model (Table 4). Concerns about physical symptoms and impacts on HRQoL expressed by participants with CFBE were consistent with the preliminary conceptual model.

Potential differences arose from three new concerns that were not explicitly mentioned in the preliminary conceptual model: hemoptysis, sinus symptoms, and control. Hemoptysis and sinus symptoms as new concerns were raised by participants in the second focus group; however, they were minority mentions (two mentions and one mention, respectively) and would thus likely not reach the point of population-level concern/impact (which would necessitate additional focus group discussions). The feeling of loss of control was mentioned three times in the first focus group.

**Table 4:** Mention counts for elements of the conceptual model

Concept	Domain	Concern	Mentions	Total
Symptom	Respiratory	Cough Sputum Dyspnea Wheeze Chest discomfort Sinus symptoms* Sputum-hemoptysis*	27 18 5 2 14 1 3	70
	Systemic	Fever/PEx Malaise/global health	21 0	21
	Fatigue	Low energy	16	16
	Sleep	Inability/disturbance	4	4
HRQoL	Physical functioning	ADLs Exercise and leisure	3 3	6
	Role functioning	Family and social Work/school Treatment burden	33 7 30	70
	Emotional functioning	Fear/anger Anxiety/depression Control*	9 43 3	55
**Grand total**				**242**

ADL, activity of daily living; HRQoL, health-related quality of life; PEx, pulmonary exacerbation

*These items were not included as specific concerns in the preliminary conceptual model prior to conducting the focus groups

Mentions/codes from the focus group were also mapped back to the granular Detailed Concerns, where HRQoL impacts were more varied. These detailed concerns appeared more closely associated with individual question items in existing PROMs (Table 5). The distribution of mentions was relatively even across the Symptom (111 mentions) and HRQoL (131 mentions) categories. Just over half (57/111) of mentions of Detailed Concerns relating to physical symptoms were classified as non-specific (not explicitly associated with the severity or frequency of a symptom). Very few (5/131) mentions of Detailed Concerns for HRQoL impacts were non-specific. Concerns with the greatest number of mentions (≥ 20 mentions) included cough (respiratory [Symptom]), exacerbations (systemic [Symptom]), family and social (role function [HRQoL]), treatment burden (role function [HRQoL]), and anxiety/depression (emotional function [HRQoL]). Of these, the concern with the highest number of descriptions was treatment burden (sub-categories included concerns related to family impacts, system of care as burden, self-care, managing treatment, and hospitalization). Concerns relating to mental health issues (anxiety, worry, depression, and isolation), however, had the highest number of mentions (43 mentions), indicating high relevance to people with CFBE.

**Table 5:** Detailed concerns: Mention counts for elements from the preliminary conceptual model

Concept	Domain	Concern	Detail 1		Detail 2		Detail 3		Detail 4		Ns		*N*
			Description	*n*	Description	*n*	Description	*n*	Description	*n*	Description	*n*	
Symptom	Respiratory	Cough	Frequency	7	Severity	14					ns	6	27
		Sputum	Frequency	2	Severity	7					ns	9	18
		Dyspnea	Frequency	1	Severity	1					ns	3	5
		Wheeze	Frequency	2	Severity	-					ns	-	2
		Chest discomfort/pain	Frequency	3	Severity	7					ns	4	14
		Sinus	Frequency	-	Severity	1					ns	-	1
		Hemoptysis	Frequency	-	Severity	1					ns	2	3
	Systemic	PEx	Frequency	2	Severity	-					ns	19	21
		Malaise/global health	Frequency	-	Severity	-					ns	-	0
	Fatigue	Low energy	Frequency	2	Severity	2					ns	12	16
	Sleep	Inability/disturbance	Frequency	-	Severity	2					ns	2	4
HRQoL	Physical functioning	ADLs	-	-	-	-					ns	3	3
		Exercise and leisure	Exercise & sports	2	Other leisure activities, vacations	1					ns	-	3
	Role functioning	Family and social	Family-specific	12	Friends & other social	20					ns	1	33
		Work/school	Ability	3	Impacts on work setting	4					ns	-	7
		Treatment burden	Family impacts	-	System of care as burden	3	Self-care, managing treatment	25	Hospitalization	1	ns	1	30
	Emotional functioning	Fear/anger	Fear & uncertainty	5	Anger	3	Frustration	1			ns		9
		Anxiety/depression	Anxiety & worry	15	Depression	22	Isolation	6					43
		Control	Loss of control	3									3
**Total**													***242***

ADL, activity of daily living; HRQoL, health-related quality of life; ns, not specified; PEx, pulmonary exacerbation

## Discussion

People with CF experience significant treatment, clinical, and psychosocial burdens. The treatment burden of respiratory-related symptoms of CF is significant, requiring patients to follow extensive treatment regimens involving numerous therapies (including antibiotics, mucolytics, and cystic fibrosis transmembrane conductance regulator [CFTR] modulators for those who are eligible) [41, 42]. Despite this extensive treatment regimen, people with CFBE are often prone to significant clinical complications, including PExs and infections, and have considerable psychosocial impacts extending to emotional well-being, mental health, interpersonal relationships, education, and careers [43, 44]. HRQoL and symptom burden are thus severely impacted in people with CFBE, and it is possible that these burdens may increase with disease progression [43].

Despite the disease experience of people with CF having been well documented, few PROMs have been developed specifically for the respiratory component of CF (CFBE) – namely, the CFQ-R Respiratory Symptoms Scale (shared by extension with the QOL-B) [30], with most CF-specific PROMs focusing on the disease in general. Although there are differences in the initiating mechanisms of NCFBE (various causes) and CFBE (CFTR dysfunction) [17, 45], the resultant BE outcome is similar. Both are characterized by dilation of the airways, impaired mucociliary clearance, and persistent infection and neutrophilic inflammation [1, 17]. As such, and despite their historical separation as distinct diseases (likely due to the pediatric focus of CF), NCFBE and CFBE share similar disease features that likely warrant the shared use of PROMs.

The concerns about physical symptoms and HRQoL impacts expressed by people with CFBE were consistent with concerns captured in existing PROMs used for BE, which were developed for or validated primarily in people with NCFBE. These results indicated substantial similarity of concerns across physical symptoms, with the most common concerns being cough, sputum, chest discomfort, and exacerbations. Hemoptysis and sinusitis were raised during the focus group discussions, but not explicitly identified in the preliminary conceptual model. However, hemoptysis is captured in several of the PROMs, including the BEST daily symptom diary [36]; additionally, chronic rhinosinusitis is prominent in BE, occurring in roughly two-thirds of adults with BE, and has also been associated with poorer HRQoL in people with BE [46].

Regarding HRQoL, Physical, Role, and Emotional functioning concerns in the preliminary conceptual model were echoed by focus group participants. The functional impacts with the greatest number of mentions were “Family and Social role functioning”, “Treatment burden”, and anxiety and depression among “Emotional functioning”. One potential difference between the CFBE focus group results and the preliminary conceptual model was in feelings of loss of control. One could hypothesize that this feeling of loss of control is likely to overlap with other concerns such as anxiety [47] and the overwhelming treatment regimen associated with CF [47, 48], both of which could possibly contribute to feelings of loss of control [49]. Although “control” as a unique concern was not included in the preliminary conceptual model, both the BIM and QOL-B do mention items that may address the concept of “control” [27, 33].

When reviewing the more granular Detailed Concerns, the results again suggested similarities in all people with BE, independent of underlying etiology. However, the results also suggest two potential differences regarding treatment burden. Focus group participants mentioned “system of care as a source of treatment burden” and, more frequently, “self-care and managing treatments”. Since these specific issues are not explicitly included in existing PROMs used in NCFBE, these concerns relating to treatment burden may illustrate a potential difference in the lived experiences between people with CF and people who developed BE due to other reasons. With 30 mentions overall, treatment burden appears to be a substantial concern in people with CFBE and may be a notable cause of difference in lived experience versus people with NCFBE.

The authors postulate that the potential differences in patient experience may stem from adaptation to respiratory diseases, given that adults with CF may have adapted to living with chronic lung disease since symptoms begin in childhood [18]. Such long-term adaptation may engineer useful coping mechanisms, greater familiarity with treatment routines, and long-established self-management strategies and relationships with healthcare providers. In contrast, many people with NCFBE may struggle adjusting to their condition and treatments, as the disease mostly arises as a new-onset condition in older adults [20, 21]. Physically, people with CFBE are already familiar with respiratory symptoms such as chronic coughing, sputum production, and infection [45, 50, 51]. However, they may face increased symptom severity, since CFBE is likely to further decrease lung function. Furthermore, adapting to the cumulative impact of additional lung conditions may heighten anxiety or depression in people with CFBE.

Limitations of this study should be considered. Firstly, our sample was fairly homogenous—the small size of the focus groups involved participants who were recruited through a single patient engagement resource, and all were from the USA. The focus groups included only two participants from the South Census region and only one non-White participant. Because of these factors, the mentioned limitations may have resulted in a population that does not fully represent the spectrum of the lived experience with CFBE, especially when considering individual disease severity and geographical heterogeneity. Additionally, participants in the study had a self-reported CFBE diagnosis (a potential risk that CFBE was not clinically confirmed in the study population). Furthermore, the focus group discussions were 2 h long, which may have caused participants to fatigue and therefore limit their participation. Another limitation inherent to focus groups is that patients may not feel comfortable sharing aspects of their disease in a group setting—a limitation that may be mitigated by personal interviews. Additionally, individuals who are naturally more introverted or anxious may be unable to discuss their concerns in a group setting without feeling overshadowed by extroverted individuals who have the confidence to speak more. Concerns surrounding “Physical function” manifestations in BE tended to focus on impacts related to daily life and routine activities; for example, most of the physical function items were generally related to jobs and the ability to work. This may have been a result of the limited probes, both in number and quality, outlined in the focus group discussion guide (Supplementary Material 2). As a result of this, it is possible that the resultant feedback from participants may have been generalized. However, while these impacts may be applicable to most chronic diseases and not specific to respiratory ones, they are nonetheless meaningful in the context of BE. Finally, because the preliminary conceptual model was based on a BE population that included CFBE (and not an NCFBE population alone), similarities in symptoms and impacts may have overlapped between the CF populations referenced in the preliminary conceptual model and the people with CFBE who participated in the focus groups. Nevertheless, a strong alignment between the preliminary framework and resultant outcomes was seen in people with CFBE.

## Conclusions

This research found that HRQoL impacts that are most relevant to people with CFBE are captured in existing multi-dimension PROMs and daily symptom diaries that were primarily developed and validated in NCFBE. The results of this research suggest that any potential differences in the lived experience between people with CFBE and NCFBE in our sample are minimal, with the exception of potential differences in perceived treatment burden. Existing PROMs primarily developed and validated in NCFBE may thus be fit for purpose in people with CFBE, pending additional cognitive and psychometric validation research. It is also advised that the selection of specific PROMs should be guided by their intended purpose.

## Electronic Supplementary Material

Below is the link to the electronic supplementary material.

Supplementary Material 1

Supplementary Material 2

Supplementary Material 3

Supplementary Material 4

## Data Availability

Data are available upon reasonable request.

## Abbreviations

BE: Bronchiectasis
BEST: Bronchiectasis Exacerbation and Symptoms Tool
BIM: Bronchiectasis Impact Measure
CAAT: Chronic Airway Assessment Test
CF: Cystic fibrosis
CFBE: Cystic fibrosis bronchiectasis
CFQ-R: Cystic Fibrosis Questionnaire – Revised
CFTR: Cystic fibrosis transmembrane conductance regulator
HRQoL: Health-related quality of life
NCFBE: Non–cystic fibrosis bronchiectasis
PEx: Pulmonary exacerbation
PROM: Patient-reported outcome measure
QOL-B: Quality of Life Questionnaire – Bronchiectasis
SGRQ: St. George’s Respiratory Questionnaire
