# Supplementary material for: Patient-reported outcome measures developed for non–cystic fibrosis bronchiectasis may be applied to cystic fibrosis bronchiectasis
Source: Health Qual Life Outcomes. 2026 May 13;24:74. doi: 10.1186/s12955-026-02546-4 (PMC13188592; doi:10.1186/s12955-026-02546-4)
Supplement: Supplementary file 1 — Supplementary Material 1 [file 12955_2026_2546_MOESM1_ESM.docx]

**Supplementary Material 1**

**Patient-reported outcome measures developed for non–cystic fibrosis bronchiectasis may be applied to cystic fibrosis bronchiectasis**

Patrick A. Flume^1^, Robert J. Nordyke^2^, Donald Han^3^, Ashok Jha^4^, Gina Nicholson^2^,
John Devin Peipert^5^

^1^Medical University of South Carolina, Charleston, SC, USA; ^2^Beta6 Consulting Group, Los Angeles, CA, USA; ^3^Boehringer Ingelheim Pharmaceuticals, Inc., Ridgefield, CT, USA; ^4^Boehringer Ingelheim International GmbH, Ingelheim am Rhein, Germany; ^5^Centre for Patient Reported Outcomes Research, University of Birmingham, Edgbaston, Birmingham, UK

**Literature review used to support the development of the preliminary conceptual model**

1) The assessment of relevant PROMs from the peer-reviewed literature

To assess relevant patient-reported outcome measures (PROMs) from peer-reviewed literature, English language-based articles in PubMed (from January 2000 to April 2022) were reviewed using the following search term: “Title/Abstract: bronchiectasis AND (patient reported OR quality of life OR HRQoL)”. Additionally, from the list generated, a search of each article’s reference list was performed to supplement the initial findings. Studies were included if they reported on a) qualitative development or psychometric validation of a PROM for bronchiectasis (BE), or b) qualitative research, focus groups, or individual interviews in people with BE.

2) The assessment of PROMs used in registered BE clinical trials

To assess PROMs used in registered BE clinical trials, the following search terms were used on the ClinicalTrials.gov website: Funder Type (industry), Study Type (interventional), Study Phase (Phase II or Phase III), Condition/Disease (bronchiectasis), and Dates (January 2000 to April 2022).

3) The summary of the prescribing information for selected analog products for severe respiratory conditions

Finally, the prescribing information from the following analog products, indicated for various severe respiratory conditions, was assessed to identify any PROMs used: Breztri (chronic obstructive pulmonary disease [COPD]), Daliresp (COPD), Dupixent (COPD, asthma, and chronic rhinosinusitis with nasal polyps), Fasenra (severe eosinophilic asthma), Spiriva (COPD and asthma), Tezspire (severe asthma), Trelegy (COPD and asthma), Trikafta (cystic fibrosis), and Yupelri (COPD).
