# Supplementary material for: Patient-reported outcome measures developed for non–cystic fibrosis bronchiectasis may be applied to cystic fibrosis bronchiectasis
Source: Health Qual Life Outcomes. 2026 May 13;24:74. doi: 10.1186/s12955-026-02546-4 (PMC13188592; doi:10.1186/s12955-026-02546-4)
Supplement: Supplementary file 2 — Supplementary Material 2 [file 12955_2026_2546_MOESM2_ESM.docx]

**Supplementary Material 2**

**Patient-reported outcome measures developed for non–cystic fibrosis bronchiectasis may be applied to cystic fibrosis bronchiectasis**

Patrick A. Flume^1^, Robert J. Nordyke^2^, Donald Han^3^, Ashok Jha^4^, Gina Nicholson^2^,
John Devin Peipert^5^

^1^Medical University of South Carolina, Charleston, SC, USA; ^2^Beta6 Consulting Group, Los Angeles, CA, USA; ^3^Boehringer Ingelheim Pharmaceuticals, Inc., Ridgefield, CT, USA; ^4^Boehringer Ingelheim International GmbH, Ingelheim am Rhein, Germany; ^5^Centre for Patient Reported Outcomes Research, University of Birmingham, Edgbaston, Birmingham, UK

**Focus group discussion guide**

**Introductory statements**

“*Thank you for joining today. The purpose of this meeting is to discuss how bronchiectasis makes you feel and how it affects your day-to-day life. I’m (NAME), and my job is to help facilitate the discussion today. As background, (GIVE VERY BRIEF BIO-Where you work, where you are from. Any info that makes you more personal to the group/connects you).*

*We’d like to run today’s discussion as a focus group, which is a standard research method and really just a bunch of people getting together to discuss how they feel about a specific topic. Researchers use focus groups to learn more about specific groups of people, and their beliefs, opinions, experiences and feelings on particular topics. In this case, you’re all people who are affected by bronchiectasis. I’ll ask a few specific questions here and there, but the main point is to get your input.*

*There are no right or wrong answers or feelings here; this is an opportunity for you to share your experiences. Everybody is different and has different experiences and feelings. Do not hesitate to speak up when you have a point you would like to make. I’d like the discussion to be informal, so there’s no need to wait for me to call on you to respond. In fact, please do talk about comments other people make, whether you feel the same way or different, and why. However, during the discussion, it is important to speak one at a time so we can hear everyone’s input. If you don’t understand a question, please let me know. I am here to ask questions, listen, and make sure everyone has a chance to share. The researchers are interested in hearing from each of you, so if we seem to be stuck on a topic, I may interrupt or change the subject. If I do, please don’t feel bad about it, it’s just my way of making sure we get through all of the questions and everyone has a chance to talk.”*

**Logistics and guidance to group**

Expectations on length of discussion and summary of topics covered.

Information on recording: Use to supplement notetaking and analysis of responses. Recording deleted once project is complete.

Intent for everyone’s voice to be heard:

- “*Best if this is truly a group discussion among you as prompted by moderator’s questions”*
- “*Don’t hesitate to interrupt the moderator to continue the discussion on a particular topic or return to a prior topic*”
- “*Again, we may call on individuals to ensure we’re hearing all perspectives*”

Completion of consent forms.

**Participant background**

“*Understanding how different patients experience bronchiectasis and the impacts that it has on your lives is critical for our clinical trials. We therefore would like to make sure that we have information on your background including (paper/email form, could be filled out beforehand):*

- *Number of years you’ve lived with bronchiectasis*
- *Cause of bronchiectasis: history of CF, Connective Tissue Disease, Immunoglobulin Deficiency, COPD, or other conditions*
- *Number of exacerbations, or episodes of clinical worsening, (self-reported; or use of Rx antibiotics) and hospitalizations in past year*
- *Current work/education status, e.g.: employed (full/part), in school, on disability (under 65), retired, etc.”*

**Lead statement and group introductions:**

Ground the discussion in “PATIENT EXPERIENCE”

*“We want to focus on the many effects that bronchiectasis has on your life. These effects will certainly include the symptoms of bronchiectasis but will also include impacts on your ability to take part in normal activities of daily life, your relationships with family and friends, how you feel emotionally, and the concerns you may have about treating and managing your condition. Sometimes it can be hard to separate these issues from the effects of other conditions you may have. Where it is clear to you – let us know; where it isn’t clear where that separation is – let us know that too.”*

Introductions: Background and patient “bottom lines”.

*“So, to start, as you introduce yourselves, could you briefly describe your background and provide just a sentence or two on how bronchiectasis affects your life; what does it mean to you?”*

**Topics and issues for discussion**

Symptoms and health-related quality of life

**1) Physical symptoms**

*“We’d like to start with the physical effects of bronchiectasis.*

*Thinking about a typical day or week, what are the main physical symptoms caused by bronchiectasis?”*

PROBES:

- “Can you tell me more about the symptoms, about the frequency, severity, and how it impacts you in your day-to-day life?”
- “Why do you think this problem is related to your bronchiectasis?”
- “*If not mentioned, what about shortness of breath, chest tightness, and wheezing*”
- “*If not mentioned, what about any appetite or digestive problems caused by bronchiectasis; are they a problem for you?*”
- “*If not mentioned, does bronchiectasis affect your sleep? How?*”
- “*If not mentioned, does bronchiectasis affect your ability to remember things or solve complex problems? How?*”

*“How would you rank the symptoms we’ve just discussed; what are the top 2 or 3 most bothersome symptoms?”*

PROBES:

- “Can you tell me more about why you selected these symptoms as the most bothersome?”

**2) Psychological symptoms and emotional impact**

*“Now, let’s turn to how bronchiectasis makes you feel inside; how it affects your emotions.*

*Thinking about a typical day or week, what are the main emotions that you attribute to bronchiectasis?”*

PROBES:

- “*What emotions do you have because of bronchiectasis? Examples if necessary: sad, angry, enthusiastic?*”
- “*Why do you think bronchiectasis made you feel (Use emotion already stated: sad, angry, or frustrated, etc.?)*”
- “*Are there any particular events, like an infection perhaps, that can really change how you feel?*”

*“How would you rank the symptoms we’ve just discussed; what are the top 2 or 3 most troubling emotional effects of bronchiectasis?”*

PROBES:

- “*Can you tell me more about why you selected these symptoms as the most bothersome?*”

**3) Family functioning**

*“Now, I would like to focus on your day-to-day life, and how bronchiectasis impacts your family life, your social life and your work life. Does bronchiectasis impact your relationship with your family; how?”*

PROBES:

- “*Can you tell me more about it?*”
- “*How do you think bronchiectasis affects your family?*”
- “*Has it affected the way you act or things you do with your family? If yes, how?*”

**4) Social functioning**

*“How has bronchiectasis affected your relationships with others and your social activities?”*

PROBES:

- “*Can you tell me more about it?*”
- “*Have your friends treated you differently? If yes, how so?*”
- “*Do you go out more or less with your friends now?*”
- “*Does bronchiectasis prohibit you from joining in on certain activities? If yes, how so, and which activities?*”
- “*How do these (social) impacts vary by disease type/history/severity [if the discussion+background information is not enough to define this topic.]*”

**5) Social functioning and work**

*“If you are/were working recently or going to school, how has bronchiectasis changed your work life or school life?”*

PROBES:

- “*Do your co-workers/classmates know about your condition? If so, what do you think your co-workers/classmates think about your condition?*”
- “*How does it influence your work? What can you do or how much? Your commute? Communication with coworkers or customers?*”
- “*How do these (social) impacts vary by disease type/history/severity [if the discussion+background information is not enough to define this topic.]*”

**6) Activities of daily living**

*“How does managing bronchiectasis affect everyday activities like cooking, running errands, doing chores and getting around the house?”*

PROBES:

- “*How does bronchiectasis impact your daily schedule?*”
- “*How does it impact your daily hygiene habits (i.e. brushing teeth, washing clothes, changing, etc.)?*”
- “*How does it impact your sleeping habits?*”
- “*Does it impact the way you run errands?*”
- “*Have you stopped participating in any hobbies because of bronchiectasis? What and why?*”
- “*What additional assistance do you need in order to do everyday activities because of your condition?*”
- “*Have you changed the way you manage your home, such as cleaning, gardening, etc.? If yes, how so?*”

**7) Wrapping up**

*“Before moving on to the second part of the interview, is there anything we’re missing regarding symptoms and effects on quality of life?”*

**Problems specifically related to exacerbations**

*“We’ve already touched on the effects of infections and exacerbations, and we’d now like to talk about those problems directly.”*

**8) Exacerbations**

*“First off, what is an ‘exacerbation’ in your experience; how would you define it?*

*What new or worsened symptoms have you experienced when you have an exacerbation? How long does it usually take for you to start feeling better; for those worsened symptoms to fade away?”*

PROBES:

- “*Can you tell me more about it? How do exacerbations affect your quality of life?*”
- “*If not already covered: do you think about exacerbations much when you’re not actually having one? Is it something you worry about?*”

**9) Hospitalizations and outpatient visits, specifically**

*“In addition to the symptoms of an exacerbation, how do any unplanned doctor visits or trips to the hospital affect your life?”*

“*Do most of your exacerbations result in a hospital stay?*”

**10) Potential reduction in exacerbations**

*“We’ve touched on this too and I’d like to ask specifically about how you would feel if you had fewer infections/admissions each year; would that improve your quality of life, how?”*

**Treatment burden**

**11) Daily treatment and management concerns**

*“This is the last topic I have…So far, we’ve talked mainly about the disease itself, but we’d now like to discuss the effects that regular/daily treatment of bronchiectasis means; how does your current treatment make you feel; how does it affect your life, both positively and negatively?”*

PROBES:

- “*Would you tell me more about this?*”
- “*Can you describe the process you go through each day? How much time does it usually take?*”
- “*Is there anything that you’d like to change about your current treatment regimen?*”
- “*On an “average day”, how much time does it normally take for you to manage your bronchiectasis treatments?*”

**12) Call for final items**

*“Before we close, I’d like to open the floor to any important topics on the effects that bronchiectasis has on your life that we haven’t covered yet.”*

**Closing activities**

*“Ok, that ends our focus group. Thank you so much for joining today and sharing your thoughts and opinions with us. If you have additional information that you did not get to say in the focus group, please feel free to contact us. As a reminder, please do not share these personal discussions with others. We want to keep your comments confidential. Thank you!”*
