# Supplementary material for: Patient-reported outcome measures developed for non–cystic fibrosis bronchiectasis may be applied to cystic fibrosis bronchiectasis
Source: Health Qual Life Outcomes. 2026 May 13;24:74. doi: 10.1186/s12955-026-02546-4 (PMC13188592; doi:10.1186/s12955-026-02546-4)
Supplement: Supplementary file 3 — Supplementary Material 3 [file 12955_2026_2546_MOESM3_ESM.docx]

**Supplementary Material 3**

**Patient-reported outcome measures developed for non–cystic fibrosis bronchiectasis may be applied to cystic fibrosis bronchiectasis**

Patrick A. Flume^1^, Robert J. Nordyke^2^, Donald Han^3^, Ashok Jha^4^, Gina Nicholson^2^,
John Devin Peipert^5^

^1^Medical University of South Carolina, Charleston, SC, USA; ^2^Beta6 Consulting Group, Los Angeles, CA, USA; ^3^Boehringer Ingelheim Pharmaceuticals, Inc., Ridgefield, CT, USA; ^4^Boehringer Ingelheim International GmbH, Ingelheim am Rhein, Germany; ^5^Centre for Patient Reported Outcomes Research, University of Birmingham, Edgbaston, Birmingham, UK

**Discussion moderator details**

The focus groups were conducted by a trained moderator (RJN [male], PhD, MS), co-moderator (GN [female], MPH), and an observer (DH [male], BSc Pharm), where RJN and GN were consultants to Boehringer Ingelheim and DH was an employee of Boehringer Ingelheim at the time of the research. RJN and GN facilitated the focus group discussions, while DH flagged any outstanding questions requiring discussion to RJN and GN. RJN and GN have prior academic training in qualitative research methods, as well as CITI certifications in human subject protection in qualitative research. DH is a pharmaceutical industry researcher with experience in patient interviews and qualitative research methods. Those present for the focus group discussions included only the moderators, the observer, and the participants. Neither the moderators nor the observer had prior contact with the focus group participants or any personal connections to any participant. The discussion guide provided basic background on the involved moderators and observer.
