# Supplementary material for: Patient-reported outcome measures developed for non–cystic fibrosis bronchiectasis may be applied to cystic fibrosis bronchiectasis
Source: Health Qual Life Outcomes. 2026 May 13;24:74. doi: 10.1186/s12955-026-02546-4 (PMC13188592; doi:10.1186/s12955-026-02546-4)
Supplement: Supplementary file 4 — Supplementary Material 4 [file 12955_2026_2546_MOESM4_ESM.docx]

**Supplementary Material 4**

**Patient-reported outcome measures developed for non–cystic fibrosis bronchiectasis may be applied to cystic fibrosis bronchiectasis**

Patrick A. Flume^1^, Robert J. Nordyke^2^, Donald Han^3^, Ashok Jha^4^, Gina Nicholson^2^,
John Devin Peipert^5^

^1^Medical University of South Carolina, Charleston, SC, USA; ^2^Beta6 Consulting Group, Los Angeles, CA, USA; ^3^Boehringer Ingelheim Pharmaceuticals, Inc., Ridgefield, CT, USA; ^4^Boehringer Ingelheim International GmbH, Ingelheim am Rhein, Germany; ^5^Centre for Patient Reported Outcomes Research, University of Birmingham, Edgbaston, Birmingham, UK

**Thematic analysis and alignment with the preliminary conceptual model**

The analysis was conducted in two main steps. Firstly, thematic analysis [1] was used to identify recurring themes from the focus group discussions [1]. Initial thematic analysis and coding of the transcripts were conducted using ATLAS.ti Web [2], followed by expert refinement. Two researchers independently conducted initial coding of transcribed interviews, where codes were added or edited as appropriate. Coding discrepancies were ultimately resolved through discussion between the two researchers. Codes were summarized into initial themes to group similar statements, and themes were then reviewed by the research team and iteratively refined by checking them against the coded quotes and data extracts. In some cases, initial themes were merged, whereas others were split, to better represent disease impacts expressed by focus group participants. This step of group reviewing ensured that themes accurately represented the data and were internally coherent yet distinct from one another.

Secondly, the themes arising from the focus groups were assessed to see whether they were captured in the preliminary conceptual model, or whether the model should be modified to capture additional BE-related symptoms and HRQoL impacts. Results from the focus groups were mapped to the preliminary conceptual model to identify any potential gaps between the HRQoL impacts most relevant to CFBE and any existing PROMs. Only mentions that were related to CFBE specifically, or BE in general (i.e. respiratory manifestations), were included in this mapping step; CF-specific concerns or CF transmembrane conductance regulator (CFTR) modulator therapy-related impacts were excluded. The assessment was primarily conducted using a three-level conceptual model: Concept/Domain/Concern. An additional level of detail (Detailed Concern) was implied by the identified themes and individual quotes, resembling the level of detail of individual question items in existing PROMs used for BE. For example, if considering the concern of “Cough”, the Detailed Concerns of frequency and severity may better differentiate between specific questions such as “Over the last 7 days, how often did you cough during the day?” versus “Over the last 7 days, how often were you annoyed by your cough?”. At this Detailed Concern level, physical symptoms were further classified based on symptom frequency or severity. Mentions of symptoms and impacts that were not more detailed than the three-level conceptual model were labeled as having an “unspecified” detail.

**REFERENCES**

1. Braun V, Clarke V. Using thematic analysis in psychology. Qual Res Psychol. 2006;3(2):77-101.

2. Atlas.ti. Home page. <https://atlasti.com/atlas-ti-web> (Accessed 20 June 2025).
